# Atmospheric CO_2_ Alters Resistance of Arabidopsis to *Pseudomonas syringae* by Affecting Abscisic Acid Accumulation and Stomatal Responsiveness to Coronatine

**DOI:** 10.3389/fpls.2017.00700

**Published:** 2017-05-16

**Authors:** Yeling Zhou, Irene Vroegop-Vos, Robert C. Schuurink, Corné M. J. Pieterse, Saskia C. M. Van Wees

**Affiliations:** ^1^Plant-Microbe Interactions, Department of Biology, Faculty of Science, Utrecht UniversityUtrecht, Netherlands; ^2^Plant Physiology, Swammerdam Institute for Life Sciences, University of AmsterdamAmsterdam, Netherlands

**Keywords:** atmospheric CO_2_, Arabidopsis resistance, ABA signaling, coronatine, stomata

## Abstract

Atmospheric CO_2_ influences plant growth and stomatal aperture. Effects of high or low CO_2_ levels on plant disease resistance are less well understood. Here, resistance of *Arabidopsis thaliana* against the foliar pathogen *Pseudomonas syringae* pv. *tomato* DC3000 (*Pst*) was investigated at three different CO_2_ levels: high (800 ppm), ambient (450 ppm), and low (150 ppm). Under all conditions tested, infection by *Pst* resulted in stomatal closure within 1 h after inoculation. However, subsequent stomatal reopening at 4 h, triggered by the virulence factor coronatine (COR), occurred only at ambient and high CO_2_, but not at low CO_2_. Moreover, infection by *Pst* was reduced at low CO_2_ to the same extent as infection by mutant *Pst cor^-^*. Under all CO_2_ conditions, the ABA mutants *aba2-1* and *abi1-1* were as resistant to *Pst* as wild-type plants under low CO_2_, which contained less ABA. Moreover, stomatal reopening mediated by COR was dependent on ABA. Our results suggest that reduced ABA levels at low CO_2_ contribute to the observed enhanced resistance to *Pst* by deregulation of virulence responses. This implies that enhanced ABA levels at increasing CO_2_ levels may have a role in weakening plant defense.

## Introduction

The atmospheric CO_2_ level has been rising at an accelerating rate since the industrial revolution. According to the Coupled Climate-Carbon Cycle Model Intercomparison Project (C^4^MIP), atmospheric CO_2_ is predicted to reach levels varying between 730 and 1020 ppm at the end of 21st century. During recent years, various Free-Air CO_2_ Enrichment (FACE) studies were conducted to assess the long-term impact of elevated CO_2_ levels on plant performance. These studies showed that elevated CO_2_ levels typically result in enhanced plant growth, decreased transpiration, and higher water use efficiency (Coleman et al., 1993; Dermody et al., 2006; Reich et al., 2006; Jain et al., 2007; Leakey et al., 2009; Wang et al., 2012; Schmid et al., 2016). In contrast, studies using reduced CO_2_ levels revealed an association with decreased photosynthesis and reduced growth (Sage and Coleman, 2001; Temme et al., 2015). Generally, different plants respond similarly to changes in atmospheric CO_2_ levels, but also variable responses depending on the genotypic differences between plant species and species ecotypes have been reported (Murray, 1995; Li et al., 2006; Temme et al., 2015). For example, levels of the major metabolites fructose, galactose, and glucose decreased significantly under elevated CO_2_ conditions in *Arabidopsis thaliana* (Arabidopsis) ecotype Cvi-0, but not in the ecotypes Col-0 and Ws-0 (Li et al., 2006).

The impact of the atmospheric CO_2_ concentration on the level of plant disease resistance is highly variable (Chakraborty et al., 2000; Garrett et al., 2006; Kobayashi et al., 2006; Yáñez-López et al., 2014). High CO_2_ concentrations increase the canopy size and leaf humidity, resulting in a microclimate that is favorable for the development of many pathogenic microbes (Manning and Tiedemann, 1995). Nevertheless, at elevated CO_2_ the infection rate of the anthracnose *Colletotrichum gloeosporioides* on the pasture *Stylosanthes scabra* was significantly reduced (Chakraborty and Datta, 2003). Intriguingly, in a FACE study assessing the effects of elevated CO_2_ on soybean diseases, it was observed that high CO_2_ increased the susceptibility to brown spot *Septoria glycines*, whereas the susceptibility to downy mildew *Peronospora manshurica* was reduced (Eastburn et al., 2010). Moreover, high CO_2_-induced susceptibility of Arabidopsis to powdery mildew (*Erysiphe cichoracearum*) was reported to be dependent on the Arabidopsis ecotype (Lake and Wade, 2009). These results indicate that the effect of atmospheric CO_2_ on disease resistance is influenced by plant genotype, pathogen species, and environmental conditions.

Stomata serve as important passages for many foliar plant pathogenic microbes to access the plant (Melotto et al., 2008; Grimmer et al., 2012). Stomata also control the exchange of gases, such as water vapor and CO_2_, between the atmosphere and the leaves, hence their formation and aperture is influenced by atmospheric CO_2_. Elevated atmospheric CO_2_ levels generally lead to a decrease in stomata density and stomatal aperture (Israelsson et al., 2006). Atmospheric CO_2_ levels also influence the opening and closure of stomata. Several molecular players have been identified in this process, including the protein kinase HT1 (HIGH LEAF TEMPERATURE1), which is a key regulator of CO_2_-induced stomatal movement, and the MATE transporter RHC1 (RESISTANCE TO HIGH CO_2_ 1), which represses HT1 (Hashimoto et al., 2006; Tian et al., 2015; Hashimoto-Sugimoto et al., 2016). In addition, carbonic anhydrases and bicarbonate have been identified as early regulators of CO_2_ signaling in Arabidopsis guard cells, as they enhance the physical interaction between RHC1 and HT1 (Hu et al., 2010; Xue et al., 2011; Tian et al., 2015). The latter process activates OST1 (OPEN STOMATA1) and SLAC1 (SLOW ANION CHANNEL1), resulting in an efflux of anions and subsequent closure of stomata (Xue et al., 2011; Tian et al., 2015), a process which is dependent on ABA signaling (Chater et al., 2015).

The effects of different CO_2_ conditions on stomata behavior potentially modify pathogen infection. At elevated CO_2_ levels, red maple leaves showed enhanced resistance to the fungus *Phyllosticta minima*, which was associated with reduced stomatal aperture (Mcelrone et al., 2005). Also in the tomato-*Pseudomonas syringae* pv. *tomato* DC3000 (*Pst*) interaction, a correlation between increased disease resistance and a reduction of stomatal aperture was observed under elevated CO_2_ conditions (Li et al., 2014). However, stomata-independent defenses controlled by plant hormones contributed to the observed enhanced resistance as well (Zhang et al., 2015). In addition, the decrease in stomatal aperture of *Medicago truncatula* by elevated CO_2_ was demonstrated to improve aphid feeding (Sun et al., 2015).

Control of stomatal aperture is a crucial aspect of the plant defense response to pathogens. Under ambient conditions, the stomata of Arabidopsis and tomato plants close actively within 1 to 2 h after infection with the bacterial pathogen *Pst*, which restricts entry of this pathogen into the leaf and, hence, limits colonization of the host tissue (Melotto et al., 2006). Nonetheless, a subsequent 2 to 3 h later, *Pst* suppresses the stomatal closure by producing the virulence factor coronatine (COR), which is a structural mimic of an isoleucine derivative of the plant hormone jasmonic acid (JA), and effectively induces stomata reopening (Melotto et al., 2006). Interestingly, many signaling components that are involved in *Pst*-induced stomatal responses, particularly the plant hormones abscisic acid (ABA), salicylic acid (SA) and JA, have also been implicated in CO_2_-induced stomatal responses (Melotto et al., 2008; Neill et al., 2008; Zeng et al., 2010; Montillet et al., 2013). This indicates that stomata act as a key checkpoint of plant defense under changing atmospheric CO_2_ conditions.

Plant hormones play pivotal roles in gene regulatory networks that control responses to biotic and abiotic stress conditions (Fujita et al., 2006). Besides SA and JA, which are two key players in plant immune signaling, other hormones such as ABA, ethylene, auxins, gibberellins and cytokinins have been implicated in defense signaling, often by modulating the SA–JA backbone of the hormone-regulated immune signaling network (Vlot et al., 2009; Robert-Seilaniantz et al., 2011; Pieterse et al., 2012). ABA can function negatively in the post-invasive defense phase through its antagonism of SA- and JA-controlled pathogen defenses (Ton et al., 2009; Pieterse et al., 2012). For example, tomato and Arabidopsis mutants that are defective in ABA signaling are less susceptible to SA-controlled hemi-biotrophic bacteria like *Pst* and JA/ethylene-controlled necrotrophic fungi like *Botrytis cinerea* (Audenaert et al., 2002; Thaler and Bostock, 2004; De Torres-Zabala et al., 2007; Liu et al., 2015). However, ABA can also function positively in plant immunity by co-regulating the pre-invasive defense phase that controls papillae formation at the site of infection and stomatal behavior (Melotto et al., 2008; Ton et al., 2009; Pieterse et al., 2012). For example, Melotto et al. (2006) found that the ABA-deficient mutant *aba3-1* was defective in stomatal closure following infection with *Pst*, suggesting that ABA signaling is required for *Pst*-induced pre-invasive stomatal defense.

Elevated CO_2_ has been shown to influence plant hormone levels and signaling. Generally, SA signaling is enhanced and JA signaling is reduced (DeLucia et al., 2012), which was demonstrated in tomato to increase resistance to *Pst* and reduce resistance to *B. cinerea* (Zhang et al., 2015). Different results on the effects of elevated CO_2_ on ABA signaling in Arabidopsis have been reported, showing a reduction in ABA content (Teng et al., 2006), but also an increase in transcript abundance of ABA-responsive genes (Li et al., 2006). It has been demonstrated that ABA signaling interacts with CO_2_ signaling in guard cells (Leymarie et al., 1998; Israelsson et al., 2006; Kim and Maik, 2010; Hubbard et al., 2012; Merilo et al., 2013, 2015). Whether CO_2_ and ABA signaling converge in controlling defense responses is unknown.

Despite growing efforts on studying plant disease resistance under high atmospheric CO_2_, the exact signaling mechanisms underlying the effects of different CO_2_ levels on plant defense remain elusive. Moreover, up to now studies on the effects of low CO_2_ on plant immune responses are scarce. Inclusion of low CO_2_ experiments could reveal effects of the steep incline in CO_2_ levels that the world has faced since the industrial revolution (Sage and Coleman, 2001). Most plants are expected to still be adapted to lower levels of atmospheric CO_2_ than the current ambient level. Using Arabidopsis-*Pst* as a model, we set out to investigate whether and how atmospheric CO_2_ affects the disease resistance to this bacterial pathogen that gains access to the plant through stomatal openings. We observed that high CO_2_-grown Arabidopsis plants exhibited enhanced susceptibility to *Pst*, whereas plants grown under low CO_2_ conditions were more resistant. The ABA content in low CO_2_-grown plants was shown to be reduced upon *Pst* infection. The role of ABA in atmospheric CO_2_-modulated disease resistance was further investigated using ABA mutants. Both ABA mutants and low CO_2_-grown wild-type plants showed attenuation of COR-triggered stomatal reopening and displayed an enhanced resistance level to *Pst*. These data suggest that the historic rise of atmospheric CO_2_ may have caused enhanced disease susceptibility to certain pathogens due to the ABA-regulated suppression of plant immunity.

## Materials and Methods

### Plant Materials and Cultivation

Seeds of *A. thaliana* accessions Col-0 and Landsberg *erecta* (L*er*-0), and mutants *aba2-1* [Col-0] (Koornneef et al., 1982), *abi1-2* [Col-0] (Gosti et al., 1999), and *abi1-1* [L*er*-0] (Koornneef et al., 1984) were sown on autoclaved river sand under ambient CO_2_ conditions (450 ppm). Two weeks later, seedlings were transferred to 60-ml pots containing a sand/potting soil mixture that was autoclaved twice for 20 min. Then, they were placed in high (800 ppm), ambient (450 ppm), or low (150 ppm) atmospheric CO_2_ conditions, where they remained for the rest of the experiment, except in the experiments shown in **Figures 3D,E** and Supplementary Figure S3, for which the seedlings continued growing for 2 more weeks under the ambient CO_2_ condition and were only after inoculation with the *Pst* bacteria or treatment with ABA placed under the different CO_2_ conditions for the remainder of the experiment. The technical specifications of the CO_2_-controlled growth chambers used in this study have been described in detail by Temme et al. (2015). Plants grew at a 10-h day at 20°C and 14-h night at 18°C cycle (350 μmol m^-2^ s^-1^) with 70% relative humidity. Plants were watered every other day and received half-strength Hoagland solution (Hoagland and Arnon, 1938) twice a week. Plants were treated when 4 weeks old in all experiments. For dry weight measurements, 10 rosettes per time point were put separately in a paper bag and dried for 3 days at 60°C.

### Cultivation of Bacteria and Bioassays

*Pst* (Whalen et al., 1991) and *Pst cor^-^* (strain DB29 of *Pst*, which is a *cmaA cfaA* double mutant; Brooks et al., 2004) were grown on KB medium (King et al., 1954) supplemented with 50 μg ml^-1^ rifampicin. To prepare inoculum, bacteria were streaked from rifampicin-selective KB agar plates and subsequently cultured in liquid KB medium in a shaker at 220 rpm at 28°C for 24 h. Bacteria were collected by centrifugation for 10 min at 1,500 × *g*, and resuspended in 10 mM MgSO_4_. For dip inoculation, the bacterial inoculum was diluted to a final concentration of 5 × 10^7^ cfu ml^-1^ of 10 mM MgSO_4_ containing 0.015% (v/v) Silwet L-77 (Van Meeuwen Chemicals, Weesp, Netherlands). For pressure infiltration, the bacterial suspension was adjusted to a concentration of 5 × 10^6^ cfu ml^-1^ (disease assay) or 1 × 10^8^ cfu ml^-1^ (ABA measurement). The abaxial side of leaves was pressure infiltrated with a needleless syringe.

To determine bacterial growth *in planta*, leaf disks of infected plants were harvested, weighed, surface sterilized in 70% ethanol for 8 s, and washed with water immediately after. Subsequently, 200 μl of 10 mM MgSO_4_ was added to the leaf disks after which they were ground thoroughly. Aliquots of 10 μl of different dilutions were plated onto KB plates containing 25 μg ml^-1^ rifampicin. After 48 h of incubation at room temperature, bacterial colonies were counted and growth of the bacteria was calculated after log-transformation of the cfu data. Eight biological replicates were included for each time point.

### Stomata Measurement

Stomatal aperture and density were measured by a modified protocol of dental resin impressions (Geisler et al., 2000). Two components of Present Light Body (Coltène, Altstatten, Switzerland) were mixed thoroughly (v/v, 1:1) and the abaxial side of the leaves was softly pressed onto the dental resin immediately after harvesting. Leaves were removed 10 min later when the mixture had hardened. Transparent nail polish was applied to the dental resin molds to create casts, which were fixed on microscope slides with Anutex modeling wax (Kemdent, Purton, Swindon, Wiltshire, UK) for further observation.

Stomata were examined using an Olympus microscope and Analysis D Olympus Software on the pictures taken. Stomatal aperture was determined by measuring the width and length of the stomata. At least six leaves were harvested for each treatment and 20–30 observations were recorded from each leaf.

### ABA Measurement and Treatment

For ABA quantification, 60–250 mg leaf material was harvested 24 h after treatments and ground to a fine powder using liquid nitrogen. ABA was extracted as described (Scala et al., 2013). Briefly, the samples were homogenized in 0.5 ml of 70% methanol using a Precellys24 tissue homogenizer (Bertin Technologies, Berlin) by shaking at 6,000 rpm for 40 s. Subsequently, the homogenates were centrifuged at 10,000 ×*g* for 20 min at 4°C. The supernatants of two extraction steps were pooled together. ABA was quantified by liquid chromatography-mass spectrometry (LC-MS) analysis on a Varian 320 Triple Quad LC-MS/MS. Endogenous ABA levels were quantified by comparing the integrated surface area from each sample with its corresponding internal standard.

To measure stomatal responsiveness to exogenously applied ABA, leaves were dipped in a solution of 15 μM ABA in 0.015% (v/v) Silwet L-77.

## Results

### Effect of High and Low Atmospheric CO_2_ Levels on Arabidopsis Growth and Stomatal Behavior

**FIGURE 1:**
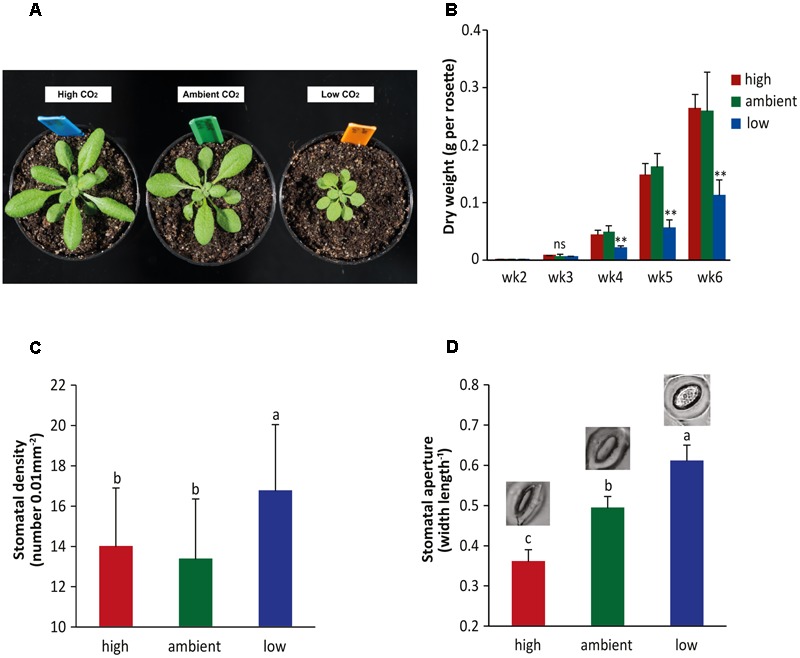
**Effect of different atmospheric CO_2_ levels on growth and stomatal behavior of Arabidopsis. (A)** Pictures of 4-week-old Arabidopsis plants grown under high (800 ppm), ambient (450 ppm), or low CO_2_ (150 ppm) conditions. **(B)** Dry weight of Arabidopsis rosettes at different developmental stages (from week 2 to week 6) under three different CO_2_ conditions. Asterisks indicate statistically significant differences between the CO_2_ treatments at the specific time points (one-way ANOVA, Duncan’s multiple range test, ^∗∗^*P* < 0.01; ns, no significant difference). Error bars represent SD, *n* = 10 plants. **(C)** Stomatal density and **(D)** stomatal aperture in 4-week-old Arabidopsis plants grown under three different CO_2_ conditions. Depicted are the averages of stomatal density and aperture (±SD) of six leaves. In **(D)** examplar pictures of stomatal aperture typical for the CO_2_ conditions are depicted. Different letters indicate statistically significant differences between the CO_2_ treatments (one-way ANOVA, Duncan’s multiple range test, *P* < 0.05). The figures are representative of at least two independent experiments.

Numerous studies have been conducted to assess the effect of high CO_2_ levels on plant performance, including plant growth, stomatal behavior, and disease resistance. However, only limited information is available on the effects of low CO_2_ levels on the plant. Here, we studied the effects of three different CO_2_ levels on Arabidopsis plants in the absence and presence of pathogens: high (800 ppm), ambient (450 ppm), and low (150 ppm) levels of CO_2_. Plants were cultivated under ambient CO_2_ until they were 2 weeks old, after which they were placed under the three respective CO_2_ conditions. We noticed that plants that had grown under the low CO_2_ condition for an additional 2 weeks had smaller rosette sizes compared to plants grown under high and ambient CO_2_ conditions (**Figure 1A**). Also the dry weight of the rosettes was significantly lower in the low CO_2_ condition (**Figure 1B**). In contrast, there were no effects on rosette growth under high CO_2_ conditions, which was rather unexpected since most previous studies have reported an increase in biomass (Bowes, 1991; Leakey et al., 2009). However, our experimental conditions may not have been optimal for stimulated growth by elevated CO_2_ (Temme et al., 2015) and moreover, the Col-0 accession that we used may respond differently to high CO_2_ than other plant species (Li et al., 2006; Leakey et al., 2009; Temme et al., 2015).

Stomatal density and aperture were investigated under the three CO_2_ conditions as well. At high atmospheric CO_2_, stomatal density was not influenced, but a significant decrease in stomatal aperture was found (**Figures 1C,D**). At low atmospheric CO_2_, an increase in both stomatal density and stomatal aperture was detected. These results are in line with previous studies that found that the inverse relationship between atmospheric CO_2_ and stomatal behavior was more evident under sub-ambient CO_2_ conditions than under elevated CO_2_ conditions (Royer, 2001), which is a phenomenon that is referred to as the CO_2_ ‘ceiling’ phenomenon. The major effects on plant growth and stomatal behavior observed at especially the low CO_2_ level prompted us to introduce a pathogen into the system that naturally enters through stomata in order to study the effects of CO_2_ on plant immunity.

### Low Atmospheric CO_2_ Inhibits COR-Triggered Stomatal Reopening

**FIGURE 2:**
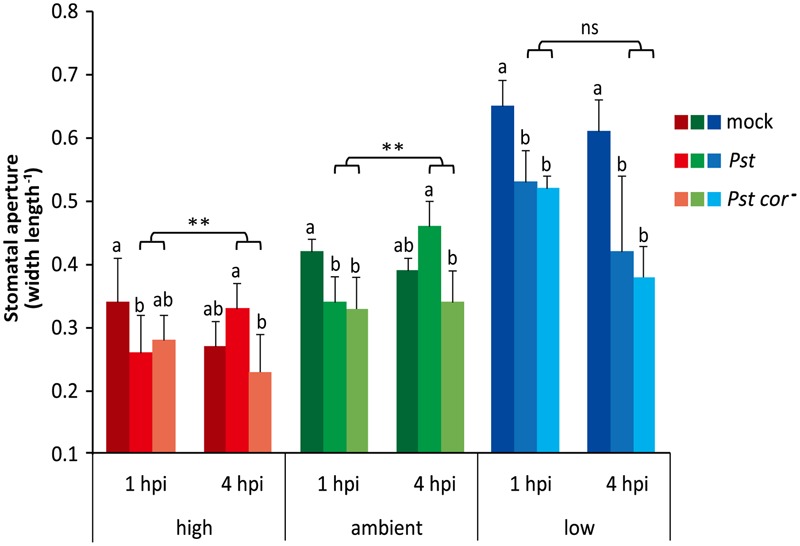
**Effect of different atmospheric CO_2_ levels on stomatal aperture upon infection by *Pst* or *Pst cor^-^*.** Arabidopsis leaves of 4-week-old plants grown under three different CO_2_ conditions were dip inoculated with a mock solution, *Pst* or *Pst cor^-^*. Stomatal aperture was determined 1 and 4 h after dip inoculation (hpi, hours post inoculation). Depicted are the averages of stomatal aperture (±SD) of six leaves. Different letters indicate statistically significant differences between the treatments at specific time points within the same atmospheric CO_2_ level (two-way ANOVA, Fisher’s LSD test, *P* < 0.05). Indications above the brackets specify the interaction (bacterium genotype × time) between the two *Pst* genotype treatments (wild type and mutant) and the time (1 and 4 hpi) under the same atmospheric CO_2_ condition (^∗∗^*P* < 0.01; ns, not significant). This figure is representative of three independent experiments.

To explore whether the differential stomatal behavior at the three tested atmospheric CO_2_ levels affects stomatal defense responses, we examined the stomatal responsiveness of Arabidopsis plants to infection by the bacterial leaf pathogen *Pst*. Previously, it was shown that at ambient CO_2_ the stomata close within 1 to 2 h after dip inoculation with *Pst*, and reopen again at 3 to 4 h due to the action of the virulence factor COR (Melotto et al., 2006). Our results under ambient conditions are in line with this finding, since we found that the stomata closed within 1 h after dip inoculation with wild-type *Pst* or the COR-deficient mutant *Pst cor^-^* (**Figure 2**). Subsequently, at 4 h after inoculation, the stomatal aperture was significantly greater in leaves infected by the wild-type *Pst* strain than by the *Pst cor^-^* mutant strain. It should be noted that for proper determination of the COR effect one should compare the mutant infection with the wild-type infection, and not with the mock treatment at 4 hpi. The circadian rhythm influences the stomatal aperture (closing in the afternoon), which may explain the somewhat more closed stomata in the mock situation at 4 hpi. Therefore, in comparison to mock, stomata of *Pst cor*^-^-infected leaves were no longer statistically significantly more closed at 4 hpi and moreover, the stomata of wild-type *Pst*-challenged leaves were not statistically significantly more open, although a trend, likely due to COR action, was visible.

High CO_2_-grown plants contained stomata that were generally more closed (**Figures 1D, 2**). Still, the stomata initially closed further when the leaves were attacked by *Pst* and near significant closure was also induced by *Pst cor^-^* infection. Similar to the ambient condition, under high CO_2_ the stomata were subsequently reopened by the wild type in comparison to the mutant bacteria. This suggests that stomatal responsiveness to PAMP-triggered closure and COR-triggered opening is intact at the high CO_2_ level. Under the low CO_2_ condition, stomata were opened more widely (**Figures 1D, 2**), but still they closed within 1 h after inoculation with *Pst* wild type or *Pst cor^-^* mutant, which is comparable to the ambient and high CO_2_ conditions. In contrast, at 4 h after inoculation, the stomata of both the *Pst*- and the *Pst cor^-^*-challenged leaves remained closed under the low CO_2_ condition. These data show that plants grown under high and low CO_2_ conditions initially respond to *Pst* infection by closing their stomata, despite their original differences in stomatal aperture. However, the subsequent COR-mediated stomatal reopening occurs only under high and ambient CO_2_ conditions, whereas it is blocked under the low CO_2_ condition.

### Atmospheric CO_2_ Alters Resistance to *Pst* in a COR-Dependent Manner

**FIGURE 3:**
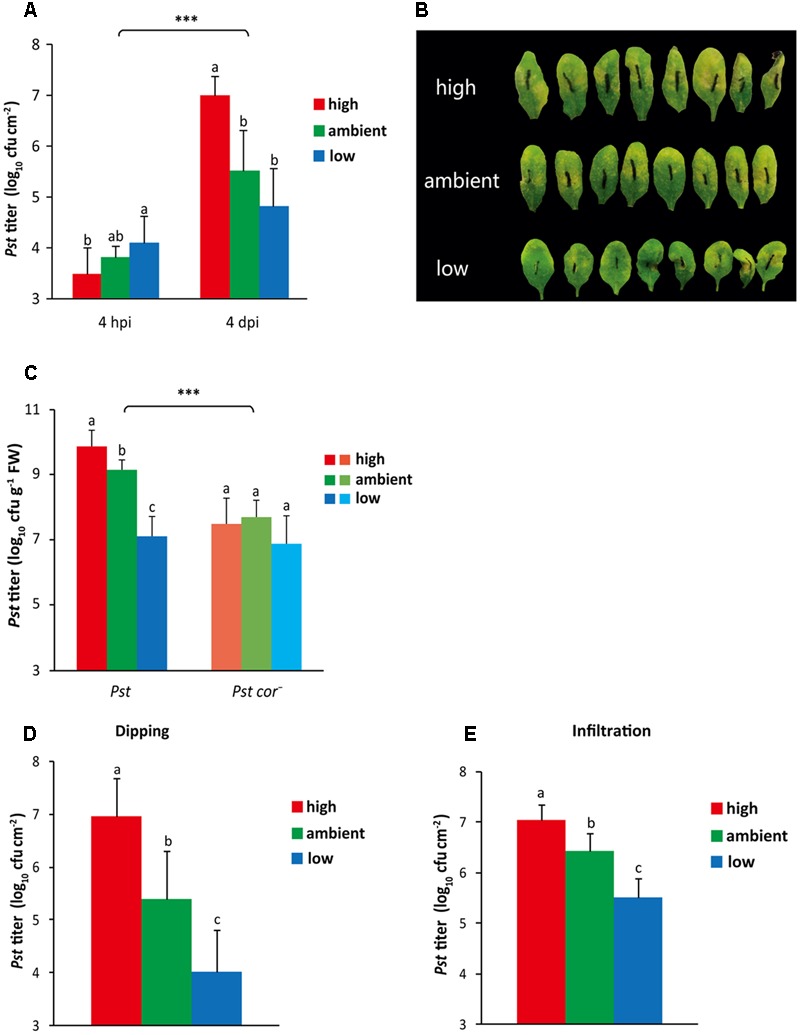
**Effect of different atmospheric CO_2_ levels on resistance of Arabidopsis to *Pst*. (A)** Growth of *Pst in planta* at 4 h and 4 days after dip inoculation of plants grown under three different CO_2_ conditions. Depicted are the averages of log_10_-transformed bacterial titer (±SD; per leaf area) from eight biological replicates. Different letters indicate statistically significant differences between the CO_2_ treatments at the indicated time point (two-way ANOVA, Fisher’s LSD test, *P* < 0.05). Indications above the brackets specify the interaction (CO_2_ condition × time) between the three CO_2_ conditions and the time (4 hpi and 4 dpi) (^∗∗∗^*P* < 0.001). **(B)** Pictures of the disease symptoms of plants grown under three different CO_2_ conditions at 4 days after dip inoculation with *Pst*. **(C)** Growth of *Pst* or *Pst cor^-^ in planta* at 4 days after dip inoculation of plants grown under three different CO_2_ conditions. Indicated are the averages of log_10_-transformed bacterial titer (±SD; per g of leaf fresh weight) from eight biological replicates. Different letters indicate statistically significant differences between the CO_2_ treatments within the same bacterial treatment (two-way ANOVA, Fisher’s LSD test, *P* < 0.05). Indications above the brackets specify the interaction (CO_2_ condition × bacterium genotype) between the three CO_2_ conditions and the two *Pst* genotype treatments (wild type and mutant) (^∗∗∗^*P* < 0.001). **(D,E)** Growth of *Pst in planta* 4 days after inoculation of plants that had been cultivated under the ambient CO_2_ condition until inoculation by **(D)** dipping (5 × 10^7^ cfu ml^-1^) or **(E)** pressure infiltration (6 × 10^5^ cfu ml^-1^), after which plants were transferred to either high, ambient, or low CO_2_ conditions. Depicted are the averages of log_10_-transformed bacterial titer (±SD; per g of leaf fresh weight) from eight biological replicates. Different letters indicate statistically significant differences between the CO_2_ treatments (Fisher’s LSD test, *P* < 0.05). These figures are representative of at least two independent experiments.

The resistance of Arabidopsis plants to *Pst* infection under the different atmospheric CO_2_ conditions was tested by determining the growth of *Pst* in plants cultivated at different CO_2_ levels. Initially, at 4 h after dip inoculation, plants grown at high CO_2_ levels contained significantly less *Pst* than plants grown at low CO_2_ (**Figure 3A**), which coincided with the lower stomatal density and aperture in leaves of high CO_2_-grown plants, thereby allowing fewer bacteria to enter the leaves (**Figures 1C,D, 2**). However, at 4 days after inoculation, the *Pst* bacterial titer in high CO_2_-grown plants was significantly higher compared to that in ambient and low CO_2_-grown plants (**Figure 3A**). In this particular experiment, the bacterial titer in low CO_2_-grown plants showed a trend of reduced amounts compared to ambient-grown plants; in other experiments the difference between the two treatments was often found to be statistically significant (**Figures 3D,E, 5B** and Supplementary Figure S2). Moreover, less-severe chlorotic disease symptoms on plants grown at low CO_2_ compared to plants grown at ambient and high CO_2_ were detected (**Figure 3B**). Given the different plant growth rates when cultivated at different CO_2_ levels, it is possible that differences in the weight per leaf area caused the detected differences in *Pst* titer when depicted per leaf area. To rule out this possibility, the bacterial titer was also determined per gram of leaf tissue. The same trend, namely enhanced multiplication of *Pst* under high, and reduced amplification under low CO_2_ conditions was found (**Figure 3C**).

The role of COR in successful infection by *Pst* through facilitation of stomatal reopening, but also by suppression of SA-mediated defense signaling and disease symptom development, has been well established (Mittal and Davis, 1995; Brooks et al., 2005). The *in planta* growth of the *Pst cor^-^* mutant strain was on average, over all three tested atmospheric CO_2_ conditions, significantly lower than that of the *Pst* wild-type strain, as was reported previously (**Figure 3C**) (Melotto et al., 2006). However, while the bacterial titer of wild-type *Pst* was significantly higher in high CO_2_-grown plants and lower in low CO_2_-grown plants, growth of the mutant *Pst cor^-^* was severely limited under all three CO_2_ conditions, reaching the same low bacterial titer as that of wild-type *Pst* in low CO_2_-grown plants (**Figure 3C**). The statistically significant interaction of atmospheric CO_2_ with the *in planta* growth difference between *Pst* and *Pst cor^-^* suggests that atmospheric CO_2_ regulates the plant’s sensitivity to COR, leading to enhanced responsiveness at high CO_2_ and impaired responsiveness at low CO_2_. This differential responsiveness to COR could play a role in the observed differences in resistance levels to *Pst* under the three tested CO_2_ conditions.

Whether the inhibition of COR-mediated stomatal reopening under the low CO_2_ condition could explain the observed high level of resistance to *Pst* was tested by assaying bacterial growth after pressure infiltration of the *Pst* bacteria into the leaves. However, due to the small leaf size of low CO_2_-cultivated plants, a different experimental set-up had to be employed in which all plants were grown at ambient CO_2_ until they were inoculated with *Pst* (when the plants were 4 weeks old), after which they were placed under either the high, ambient, or low CO_2_ condition for the remainder of the experiment. First, we tested in this set-up the effect of different CO_2_ levels on *Pst* growth after dip inoculation and found that the bacterial growth was affected similarly to the original set-up in which plants had experienced the different CO_2_ conditions already 2 weeks preceding the dip inoculation (**Figures 3A,D**). Also upon pressure infiltration of *Pst* into the leaves, the effects of CO_2_ on bacterial growth were very much alike: high CO_2_ caused enhanced susceptibility whereas low CO_2_ caused reduced susceptibility (**Figure 3E**). This suggests that under the low CO_2_ condition not only COR-mediated stomatal reopening is affected, but also the suppression of post-invasive defense responses is reduced. On the other hand, under the high CO_2_ condition post-invasive defense appears stronger downregulated.

### A Role for ABA Signaling in COR-Mediated Stomatal Reopening

**FIGURE 4:**
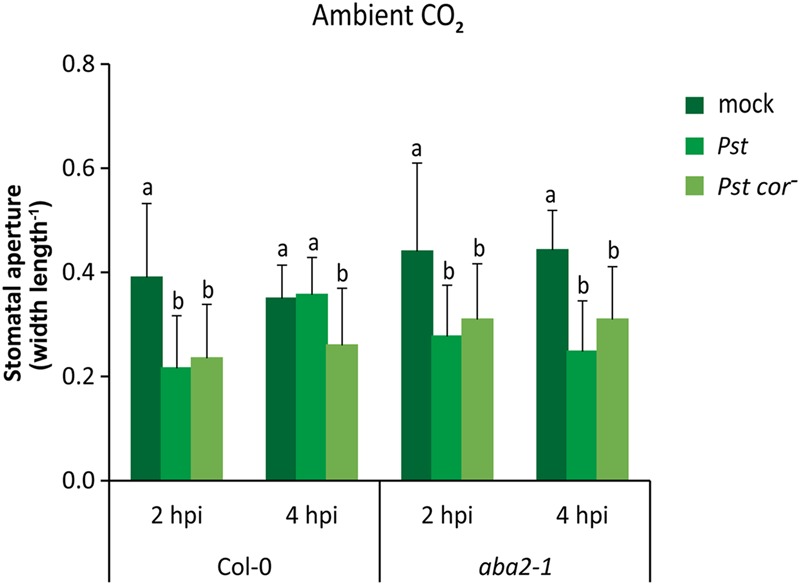
**Effect of ABA signaling on stomatal aperture in response to *Pst* and *Pst cor^-^* under ambient CO_2_ conditions.** Stomatal aperture in wild-type Col-0 and the ABA deficient mutant *aba2-1* at 2 and 4 h after dip inoculation with *Pst* or *Pst cor^-^*. Indicated are the averages of the stomatal aperture (±SD) of six leaves. Different letters indicate statistically significant differences between treatments within one plant genotype at the indicated time point (two-way ANOVA, Fisher’s LSD test, *P* < 0.05). The interaction (bacterium genotype × time) between the two *Pst* genotype treatments (wild type and mutant) and the time (1 and 4 hpi) in the same plant genotype was 0.26 for wild-type Col-0 and 0.95 for *aba2-1*. This figure is representative of two independent experiments.

**FIGURE 5:**
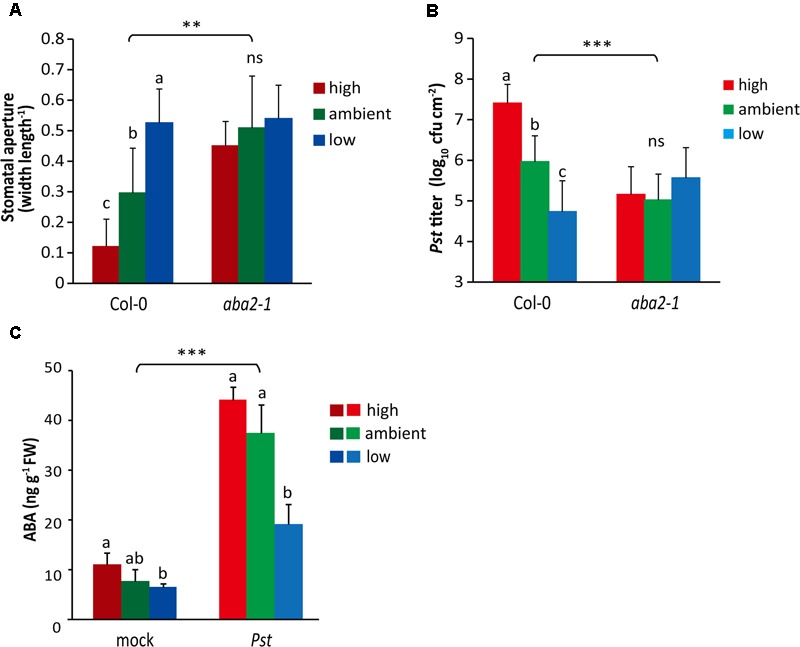
**The effect of ABA signaling on atmospheric CO_2_-altered stomatal aperture and resistance to *Pst*. (A)** Stomatal aperture of Arabidopsis wild-type Col-0 and the ABA deficient mutant *aba2-1* grown under different atmospheric CO_2_ conditions. Depicted are the averages of the stomatal aperture (±SD) of six leaves. Different letters indicate statistically significant differences between the CO_2_ treatments within the same genotype (two-way ANOVA, Fisher’s LSD test, *P* < 0.05; ns, not significant). Indications above the brackets specify the interaction (CO_2_ condition × Arabidopsis genotype) between the three CO_2_ conditions and the two Arabidopsis genotype (wild-type Col-0 and mutant *aba2-1*) (^∗∗^*P* < 0.01). This experiment has not been repeated. **(B)** Growth of *Pst* in wild-type Col-0 and the mutant *aba2-1* at 4 days after dip inoculation. Indicated are the averages of the log_10_-transformed bacterial titer (±SD; per leaf area) from eight biological replicates. Different letters indicate a statistically significant difference between the CO_2_ treatments within the same genotype (two-way ANOVA, Fisher’s LSD test, *P* < 0.05; ns, not significant). Indications above the brackets specify the interaction (CO_2_ condition × Arabidopsis genotype) between the three CO_2_ conditions and the two Arabidopsis genotypes (wild-type Col-0 and mutant *aba2-1*) (^∗∗∗^*P* < 0.001). **(C)** Levels of ABA in leaves of wild-type Col-0 plants grown under three different atmospheric CO_2_ conditions. Leaves of 4-week-old plants were pressure infiltrated with *Pst* (1 × 10^8^ cfu ml^-1^) or mock (10 mM MgSO_4_) solution and after 24 h assayed for ABA content. Indicated are the averages of ABA levels (±SD) from five biological replicates. Different letters indicate a statistically significant difference in ABA levels between the CO_2_ conditions within the same treatment (two-way ANOVA, Fisher’s LSD test, *P* < 0.05). Indications above the brackets specify the interaction (CO_2_ condition × bacterium treatment) between the three CO_2_ conditions and the treatments (*Pst* and mock) (^∗∗∗^*P* < 0.0001). The part labels **(B)** and **(C)** are representative of two independent experiments.

To gain more insight into how the differential responsiveness to COR observed under different atmospheric CO_2_ levels may alter plant immunity, we assessed the role of the hormone ABA, a known regulator of stomatal aperture, in *Pst*-triggered stomatal closure and subsequent reopening (Melotto et al., 2006). First, we studied the effect of ABA on stomatal behavior under our ambient CO_2_ condition. It has previously been shown that in non-induced situations the stomata of the Arabidopsis ABA-deficient mutant *aba2-1* and the ABA-insensitive mutant *abi1-1* can be more open than that of wild-type plants, but this effect is not always evident (**Figures 4, 5A**, and Supplementary Figure S1) (Merlot et al., 2002; Melotto et al., 2006), which may be related to difference in stomatal aperture of wild-type plants at different times of the day on which the experiments were executed (Somers et al., 1998; Dodd et al., 2004). Nevertheless, in all experiments challenge with *Pst* triggered initially stomatal closure in wild type as well as *aba2-1* and *abi1-1* plants within 1 h after dip inoculation (**Figure 4** and Supplementary Figure S1a). Moreover, while stomata subsequently reopened in a COR-dependent manner in wild-type plants as detected at 4 h after inoculation, stomata did not reopen in the *aba2-1* and *abi1-1* mutants when treated with either *Pst* or *Pst cor^-^* (**Figure 4** and Supplementary Figure S1a). These findings are in contrast with those described by Melotto et al. (2006) who showed that neither the ABA-deficient mutant *aba3-1*, nor the ABA-insensitive mutant *ost1-2*, responded with stomatal closure to infection by *Pst* or treatment with flg22, the active epitope of bacterial flagellin. However, in accordance with our results, Montillet et al. (2013) reported that mutants *aba2-1* and *ost1-2* did close their stomata upon flg22 treatment. These results suggest the existence of an ABA-independent pathway in guard cells involving pathogen-induced stomatal closure. Furthermore, our data reveal a role for ABA signaling in COR-mediated stomatal reopening.

### ABA-Dependency of Atmospheric CO_2_-Controlled Stomatal Aperture and Disease Resistance against *Pst*

Based on our finding that under ambient CO_2_ conditions ABA mutants show a stomatal response pattern to *Pst* infection that is similar to that of wild-type Arabidopsis plants grown under low CO_2_ conditions, we hypothesized that there could be a role for ABA signaling in atmospheric CO_2_-altered disease resistance to *Pst*. To test this, we first measured stomatal aperture of the mutant *aba2-1* when cultivated at different levels of atmospheric CO_2_ without *Pst* infection. As expected, stomata of wild-type Col-0 plants were more closed at high CO_2_ and more opened at low CO_2_ (**Figure 5A**). However, stomata of the *aba2-1* mutant did not change their aperture under different CO_2_ conditions. In fact, their stomata were relatively open under all three CO_2_ conditions, to the same high level as that of low CO_2_-grown wild-type plants (**Figure 5A**). This statistical interaction between ABA and CO_2_ levels suggests that ABA can be involved in stomatal responsiveness to different atmospheric CO_2_ conditions.

Subsequently, we tested whether atmospheric CO_2_ can alter disease resistance to *Pst* in ABA mutants. Under ambient and high CO_2_ conditions, both *aba2-1* and *abi1-1* exhibited reduced *in planta* growth of *Pst* compared with wild-type plants (**Figure 5B** and Supplementary Figure S2), supporting a negative role for ABA signaling in the defense response against *Pst*, as shown previously (Melotto et al., 2006; De Torres-Zabala et al., 2007). More importantly, under all three CO_2_ conditions *Pst* growth in both ABA mutants was as low as in the wild-type plants grown at low CO_2_ (**Figure 5B** and Supplementary Figure S2). Together, these results suggest that ABA signaling plays an important role in atmospheric CO_2_-regulated plant defense responses against *Pst*.

Previously, ABA has been reported to accumulate upon *Pst* infection (De Torres-Zabala et al., 2007). Moreover, enrichment in atmospheric CO_2_ can also change ABA levels or ABA signaling, although variable effects in Arabidopsis have been described (Li et al., 2006; Teng et al., 2006). We assayed the ABA content in leaves infected with *Pst* under different atmospheric CO_2_ conditions. In the absence of *Pst*, ABA accumulation under the low CO_2_ condition was significantly reduced compared to that under the high CO_2_ condition, but no statistically significant differences with the ambient CO_2_ condition were detected (**Figure 5C**). In *Pst*-challenged leaves the ABA concentrations rose significantly compared with mock-treated leaves under all three CO_2_ conditions. However, in the low CO_2_-grown plants the ABA levels were significantly lower than those in the ambient and high CO_2_-grown plants upon infection by *Pst*. These results suggest that reduced ABA levels in low CO_2_-grown plants may be responsible for enhanced resistance to *Pst*.

## Discussion

As one of the major characteristics of global climate change, the continuously rising atmospheric CO_2_ concentration has received extensive attention during the past decades. Here, we investigated the interplay between atmospheric CO_2_ and Arabidopsis defense mechanisms to infection by *Pst*. Plants are likely still evolutionary adapted to pre-industrial CO_2_ levels that are lower than the current global CO_2_ concentration. Therefore, by comparing three conditions, namely low (150 ppm), ambient (450 ppm), and high (800 ppm) CO_2_ levels, we compared the effect of the historic and future incline in CO_2_ level. Up to now studies on low CO_2_ effects on plant performance and plant disease resistance have been scarce (Tissue and Lewis, 2012). We show that effects of atmospheric CO_2_ on ABA signaling may account for the observed differential stomatal responsiveness as part of the altered resistance level to pathogen infection, and that post-invasive defenses are modulated by atmospheric CO_2_ as well.

### Plant and Stomata Performance under Low and High CO_2_ Conditions

Previous studies, focusing mainly on the effects of elevated atmospheric CO_2_, showed that growth of various plant species was promoted by high CO_2_ levels and inhibited by low CO_2_ levels (Leakey et al., 2009; Eastburn et al., 2010; Temme et al., 2015). In our study, we show that the low CO_2_ condition significantly reduced growth of Arabidopsis and caused the stomata to be opened more widely than under ambient CO_2_ (**Figure 1**). Reduced ABA content and sensitivity in low CO_2_-grown plants (**Figure 5C** and Supplementary Figure S3) may be causal for the enhanced stomatal opening phenotype, and although a reduced plant stature has been reported for ABA mutants as well (Chatfield et al., 2000; LeNoble et al., 2004), the growth reduction at low CO_2_ is more likely caused by reduced photosynthesis. We found that Arabidopsis grown at high CO_2_ displayed a reduced opening of their stomata, as has been reported previously (Araújo et al., 2011). However, we found no reduction in stomatal density, nor an increase in rosette dry weight under the high CO_2_ condition. No or small effects of high CO_2_ on growth enhancement has previously been reported (Li et al., 2006; Temme et al., 2015). In our experimental set-up light intensity and nutrient constraints may have limited the stimulating effect of high CO_2_ on plant growth. Our findings on stomatal density are in line with the previously described CO_2_ ‘ceiling’ phenomenon, which refers to reaching a maximum stomatal density at a CO_2_ level of 400 ppm and that stomata respond more strongly to sub-ambient than to elevated CO_2_ concentrations (Kürschner et al., 1997; Royer, 2001).

### *Pst*-Induced Stomatal Closure Can Be Independent of ABA and Occurs Independently of the CO_2_ Condition

Besides CO_2_, ABA determines the stomatal aperture. Here, we provide evidence for a role of ABA signaling in the regulation of stomatal aperture by different CO_2_ levels in un-infected plants. Unlike the wild type, the stomatal aperture of the ABA biosynthesis mutant *aba2-1* was not influenced by the CO_2_ conditions (**Figure 5A**). Moreover, the ABA signaling mutant *abi1-1* was unresponsive to high CO_2_-induced stomatal closure, albeit sensitivity regarding low CO_2_-induced opening was observed (Supplementary Figure S1). In general, our data corroborate previous findings on the interrelationship of ABA with elevated CO_2_-regulated signaling in guard cells (Leymarie et al., 1999; Nishimura et al., 2010; Xue et al., 2011; McLachlan et al., 2014).

Activation of stomatal closure has been demonstrated to be an essential pre-invasive defense response to foliar pathogens in various plant species (Melotto et al., 2006; Lee et al., 2013; Li et al., 2013; Du et al., 2014). The ABA-deficient mutant *aba3-1* was previously shown to be compromised in its ability to close its stomata in response to *Pst* infection, suggesting a requirement for ABA biosynthesis in *Pst*-induced stomatal closure (Melotto et al., 2006). However, in our experiments, at all CO_2_ levels tested, both wild-type plants and the ABA mutants *aba2-1* and *abi1-1* responded to *Pst* infection with closure of their stomata (**Figure 4** and Supplementary Figure S1). This indicates that the *Pst*-induced stomatal closure occurs at least partly independently of ABA and that atmospheric CO_2_ does not influence this mechanism. This is in line with a recent finding that an ABA-independent oxylipin pathway is responsible for flg22- and *Pst*-induced stomatal closure (Montillet et al., 2013). Moreover, a genetic screen of Arabidopsis mutants that are impaired in *Pst*-induced stomatal closure generated multiple mutants that still exhibited ABA-induced stomatal closure (Zeng et al., 2011). In addition, Lim et al. (2014) demonstrated that ABA hyposensitive PP2CA1 overexpressors closed their stomata in response to *Pst* infection or flg22 treatment. Taken together, these results support the notion that besides ABA signaling, additional mechanisms that are independent of ABA and independent of atmospheric CO_2_ play a role in the stomatal closure response upon *Pst* infection.

### COR-Induced Stomatal Reopening Is Blocked at Low CO_2_ and Depends on ABA

The phytotoxin COR that is produced by *Pst* induces stomatal opening to stimulate infection. We found that under both ambient and high CO_2_ conditions, initial *Pst*-induced stomatal closure was followed by COR-dependent stomatal reopening (**Figure 2**). Interestingly, while stomata in low CO_2_-grown plants still responded to *Pst* with closing within 1 hpi, they did not reopen at 4 hpi, resulting in a stomatal aperture very similar to that of *Pst cor^-^*-infected plants (**Figure 2**). Thus, if COR production by *Pst* is not affected by the low CO_2_ level, sensitivity to COR in terms of stomata reopening seems compromised under the low CO_2_ condition.

Previous reports demonstrated that under ambient CO_2_ conditions, COR and ABA signaling can influence each other’s activity either negatively or positively. For instance, ABA-induced stomatal closure is inhibited by COR (Melotto et al., 2006; Zheng et al., 2012), while both COR and ABA repress SA-regulated defense signaling (Brooks et al., 2005; De Torres-Zabala et al., 2007). Also, ABA and COR both activate gene expression of three NAC transcription factors that suppress *Pst*-induced SA biosynthesis and stomatal closure (Zheng et al., 2012). Here, we show that the mutants *aba2-1* and *abi1-1* closed their stomata upon inoculation with *Pst* and were unable to reopen them in response to COR production by *Pst* at 4 hpi under all three CO_2_ conditions tested (**Figure 4** and Supplementary Figure S1). This pointed to an unexpected role for ABA signaling in COR-mediated stomatal reopening, which is independent of CO_2_ levels. As a follow-up experiment, expression of the COR-inducible, ABA-dependent NAC transcription factor genes *ANAC019, ANAC055*, and *ANAC072* (Zheng et al., 2012) could be assessed in *aba2-1* and *abi1-1*; their reduced expression may contribute to the ABA-dependency of COR-mediated stomatal reopening. It has been demonstrated that reactive oxygen species (ROS) act as essential second messengers in Arabidopsis guard cells, functioning in CO_2_- and ABA-induced stomatal closure (Pei et al., 2000; Mustilli et al., 2002; Chater et al., 2015). Interestingly, it has recently been reported that COR inhibited ROS production in guard cells, thereby aiding the inhibition of stomatal closure (Toum et al., 2016). Whether ROS production may act as a point of convergence between CO_2_, ABA, and COR signaling and in doing so determines the outcome of COR responsiveness in ABA mutants and under different CO_2_ conditions is an important question.

### Low CO_2_ and Defective ABA Signaling Enhance Resistance to *Pst*, While High CO_2_ Reduces Resistance

In accordance with the blocked COR-induced stomatal reopening, low CO_2_-grown plants exhibited significantly reduced growth of *Pst* at 4 dpi compared with ambient CO_2_-grown plants (**Figures 3C–E, 5B** and Supplementary Figure S2). However, this decrease in susceptibility to *Pst* was apparent both in dip-inoculated and pressure-infiltrated leaves (**Figures 3D,E**), suggesting that the CO_2_ effect on *Pst* infection is beyond the interference of CO_2_ with stomatal defenses. The *in planta* growth of *Pst* under the low CO_2_ condition was arrested to the same level as that of *Pst cor^-^* under low, ambient or high CO_2_ conditions (**Figure 3C**). This demonstrates that impairment of COR-mediated defense suppression that is apparent under low CO_2_ conditions severely reduces the virulence of *Pst*. It is known that SA plays an essential role in the defense response of Arabidopsis against *Pst*. By acting as a structural JA mimic, COR triggers a signaling cascade that counteracts SA-dependent defenses, thus promoting susceptibility to *Pst* infection (Zheng et al., 2012). It is possible that under low CO_2_ conditions the function of COR is impaired, which alleviates the suppression of downstream SA signaling, resulting in enhanced resistance to *Pst*.

The inability of the mutants *aba2-1* and *abi1-1* to respond with stomatal reopening to COR, was associated with enhanced resistance to *Pst* (**Figure 5B** and Supplementary Figure S2), indicating the important role of ABA signaling in suppression of defenses by COR. The link with defective responsiveness to COR and enhanced resistance has been shown previously, albeit the role of ABA herein was in closing instead of reopening of the stomata (Lim et al., 2014). Interestingly, the *aba2-1* and *abi1-1* mutants were under all three CO_2_ conditions as resistant to *Pst* as wild-type plants grown at low CO_2_ (**Figure 5B** and Supplementary Figure S2) and *Pst* and *Pst cor^-^* grew to a similar level in the *aba2-1* mutant (data not shown). Thus, the resistance phenotype of low CO_2_-grown wild-type plants resembles that of the ABA mutants. Remarkably, also plant growth is inhibited by both low CO_2_ and ABA-related mutations (**Figures 1A,B**) (Chatfield et al., 2000; LeNoble et al., 2004). Moreover, in low CO_2_-grown plants the ABA levels were induced to a lower extent by *Pst* infection than in ambient or high CO_2_-grown plants and a trend of reduced ABA content was already visible in the non-infected situation (**Figure 5C**). In addition, responsiveness to ABA was affected under low CO_2_, shown by a significantly smaller effect on stomatal closure induced by exogenous application of ABA than under the high and ambient CO_2_ conditions (Supplementary Figure S3). Altogether, these results suggest that the enhanced resistance to *Pst* that is evident under the low CO_2_ condition is related to a decrease in ABA content and signaling.

In addition to a role in COR-triggered stomatal reopening that we demonstrated, ABA is also known to suppress SA defense signaling, possibly in part by activation of the three above-mentioned NAC transcription factors (De Torres-Zabala et al., 2007; Zheng et al., 2012). Modulation of ABA signaling by atmospheric CO_2_ may affect expression of SA-mediated defense responses. Not only did we show that the ABA mutants *aba2-1* and a*bi1-1* were more resistant to *Pst* infection, we also demonstrated that the ABA hypersensitive mutant *abi1-2* is more susceptible to *Pst* (Supplementary Figure S4). At high CO_2_ levels, wild-type Arabidopsis plants were also more susceptible to *Pst* (**Figures 3, 5B** and Supplementary Figure S2). We did not detect a significant increase in ABA content under high CO_2_, although a trend was visible (**Figure 5C**). Under high CO_2_ conditions, stomatal aperture was decreased, causing fewer *Pst* bacteria to enter the leaves, but still at 4 dpi higher bacterial titers were measured, and also upon pressure infiltration enhanced *Pst in planta* growth was detected (**Figures 1C, 3**). It is unclear how high CO_2_ interferes with plant defense responsiveness. Besides an effect on plant defense, the enhanced *Pst* growth may be caused by a favorable endophytic environment for the bacteria in terms of nutrition and water availability in high CO_2_-grown plants (Lake and Wade, 2009; Pangga et al., 2011), but this enriched condition was unlikely established within the 4 days time frame in which the plants were transferred from ambient to high CO_2_ condition, which was used for some of the experiments (**Figures 5D,E**). Moreover, in contrast to our findings, tomato plants were reported to exhibit reduced infection by *Pst* at elevated CO_2_ levels (Li et al., 2014). Thus, enrichment of the endophytic environment alone unlikely explains the full effect of high CO_2_ on enhanced *Pst* growth in Arabidopsis that we demonstrated. Possibly, the difference in genetic make-up between Arabidopsis and tomato plants can explain the difference in effect of high CO_2_ on susceptibility to *Pst*.

## Conclusion

Our results show that atmospheric CO_2_ influences resistance of Arabidopsis to *Pst*, whereby pre-industrial, low CO_2_ levels lead to an increase in resistance. ABA signaling is demonstrated to be a regulator of COR-mediated stomatal reopening and susceptibility to *Pst*. Under low CO_2_ conditions ABA levels are reduced, which could explain the defect in COR-mediated stomatal reopening and the enhanced resistance to *Pst*. The global rise in atmospheric CO_2_ may be causal for the detected increase in ABA content of plants grown under ambient compared to the low CO_2_ condition when infected by the *Pst* pathogen. Further research could aid in developing efficient strategies to maintain agricultural production.

## Author Contributions

YZ, CP, and SVW planned and designed the research. YZ conducted the laboratory work. YZ, CP, and SVW analyzed and interpreted the data and wrote the manuscript. IV-V contributed to data analysis and improved the manuscript. RS provided hormone analysis.

## Conflict of Interest Statement

The authors declare that the research was conducted in the absence of any commercial or financial relationships that could be construed as a potential conflict of interest.
