# Supplementary material for: Atmospheric CO2 Alters Resistance of Arabidopsis to Pseudomonas syringae by Affecting Abscisic Acid Accumulation and Stomatal Responsiveness to Coronatine
Source: Front Plant Sci. 2017 May 16;8:700. doi: 10.3389/fpls.2017.00700 (PMC5432532; doi:10.3389/fpls.2017.00700)

Supplemental Information

Article title: Atmospheric CO_2 alters resistance of Arabidopsis to *Pseudomonas syringae* by affecting abscisic acid accumulation and stomatal responsiveness to coronatine

Authors: Yeling Zhou, Irene Vroegop-Vos, Robert C Schuurink, Corné MJ Pieterse and Saskia CM Van Wees

Fig. S1: Effect of ABA signaling on stomatal aperture in response to *Pst* and *Pst cor*$^-$ under three CO_2 conditions.

Fig. S2: Effect of ABA signaling on atmospheric CO_2-altered resistance to *Pst*.

Fig. S3: Effect of atmospheric CO_2 on ABA-induced stomatal closure.

Fig. S4: The role of ABA signaling in Arabidopsis resistance to *Pst*.

Fig. S1: Effect of ABA signaling on stomatal aperture in response to *Pst* and *Pst cor*[-] under three different CO_2 conditions. Arabidopsis leaves of wild-type Ler-0 and ABA insensitive mutant *abi1-1*, cultivated under ambient **(S1a)**, high **(S1b)**, and low CO_2 **(S1c)** conditions, were dip-inoculated with a mock solution, *Pst* or *Pst cor*[-] (5×10^7 cfu/ml). Stomatal aperture was measured at 1 h and 4 h after dip inoculation. Depicted are the averages of stomatal aperture (±SD) of six leaves. Different letters indicate statistically significant differences between the treatments of one plant genotype at the indicated time point (two-way ANOVA, Fisher's LSD test, $P<0.05$; ns, not significant). Indications above the brackets specify the interaction (bacterium genotype × time) between the two *Pst* genotype treatments (wild-type and mutant) and the time (1 hpi and 4 hpi) within the same Arabidopsis genotype (ns, not significant). Fig. S1a is representative of two independent experiments; the experiments depicted in Fig. S1b and S1c have not been repeated.

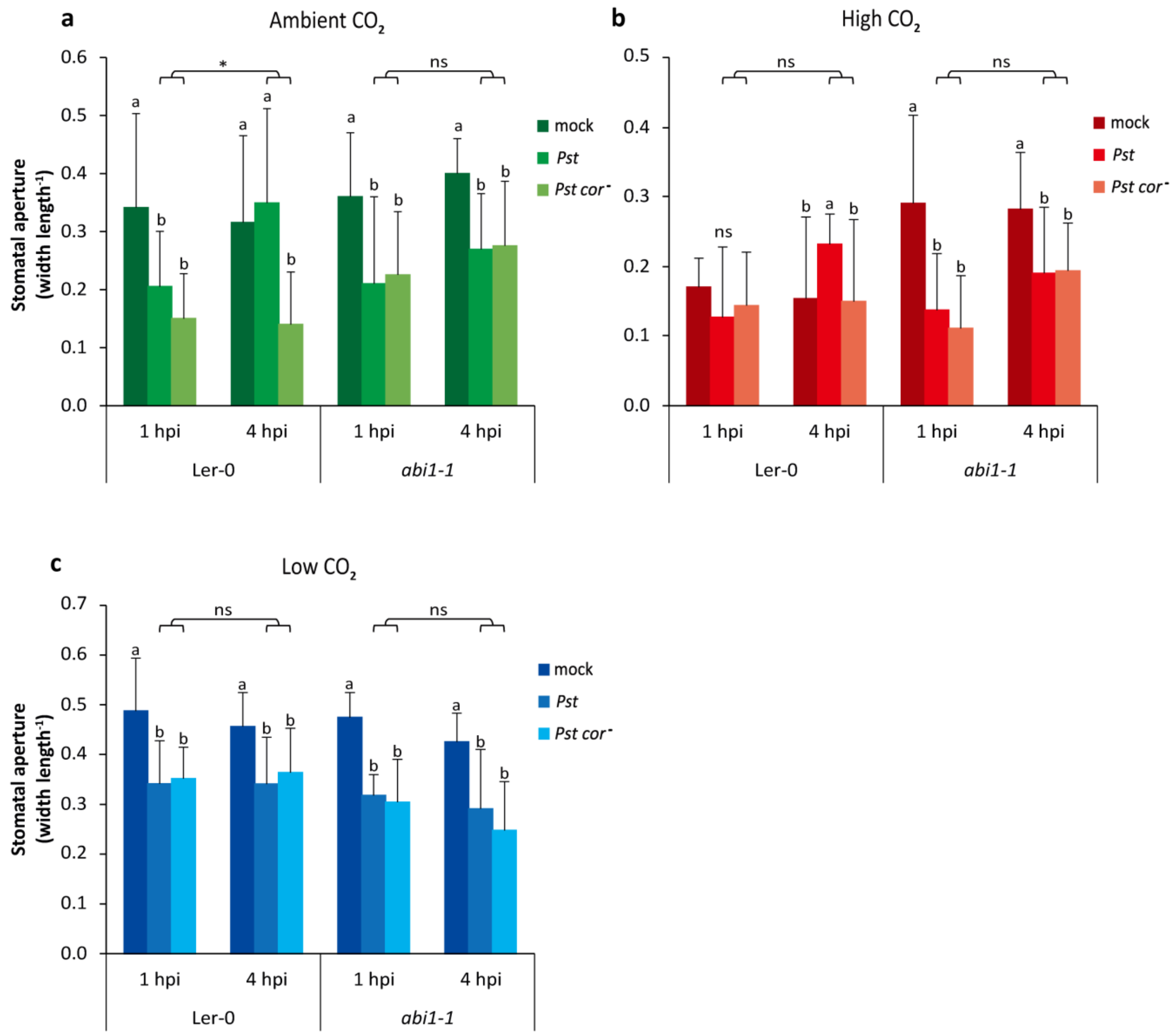

Fig. S2: Effect of ABA signaling on atmospheric CO_2-altered resistance to *Pst*. Growth of *Pst* in wild-type Ler-0 and the mutant *abi1-1* measured at 2 days and 4 days after dip inoculation. Indicated are the averages of the log_{10}-transformed bacterial titer (±SD; per leaf area) from eight biological replicates. Different letters indicate statistically significant differences between the CO_2 treatments within one line at the indicated time point (two-way ANOVA, Fisher's LSD test, $P<0.05$; ns, not significant). Indications above the brackets specify the interaction (CO_2 condition × Arabidopsis genotype) between the three CO_2 conditions and the two Arabidopsis genotype (wild-type Ler-0 and the mutant *abi1-1*) at the same time point (**, $P<0.01$; ***, $P<0.001$). The figure is representative of two independent experiments.

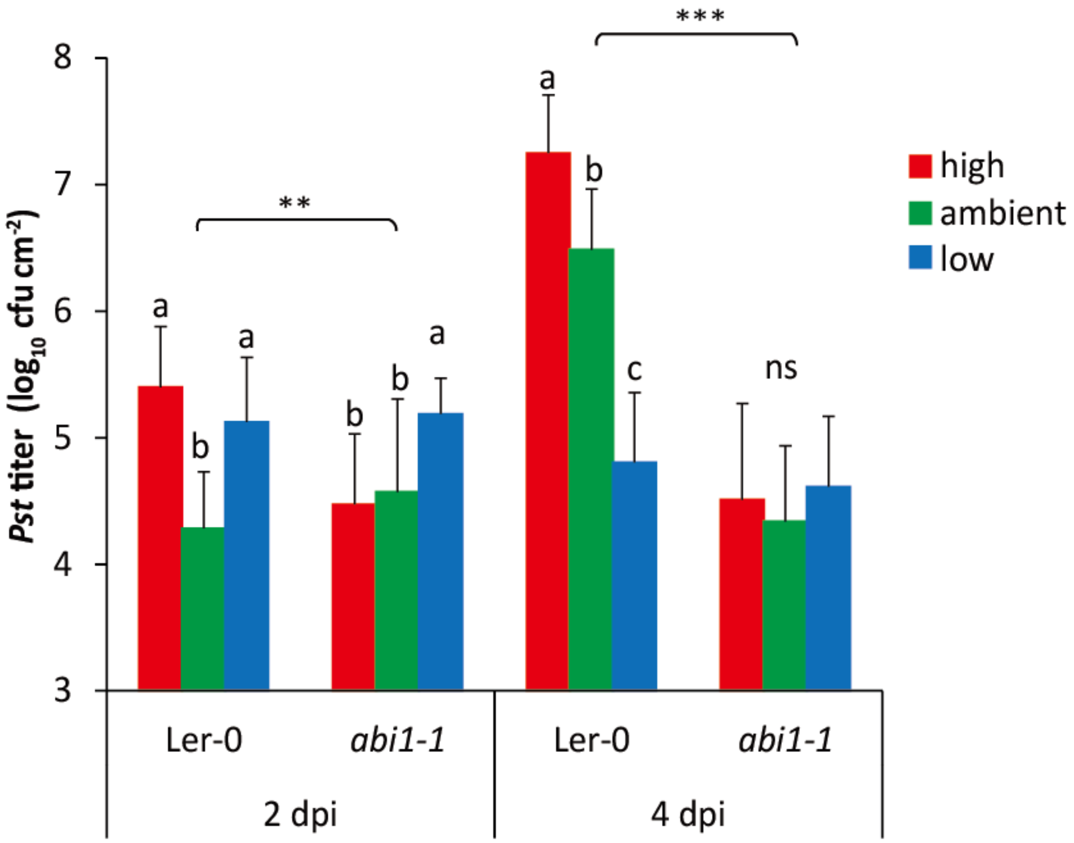

Fig. S3: Effect of atmospheric CO_2 on ABA-induced stomatal closure. Leaves of 4-week-old Arabidopsis Col-0 plants, cultivated until treatment under the ambient CO_2 condition, were exogenously supplied with a mock solution or ABA (15 µM), after which the plants were transferred to either high, ambient or low CO_2 conditions. Stomatal aperture was determined 4 h after treatment. Depicted are the averages of stomatal aperture (±SD) of six leaves. Asterisks indicate statistically significant differences between the treatments within the same atmospheric CO_2 level (Student's *t*-test, ***, $P<0.0001$, **, $P<0.001$). The asterisk above the bracket indicates there is a statistically significant interaction between the three CO_2 conditions and the treatment (two-way ANOVA, Fisher's LSD test, $P<0.05$). The figure is representative of two independent experiments.

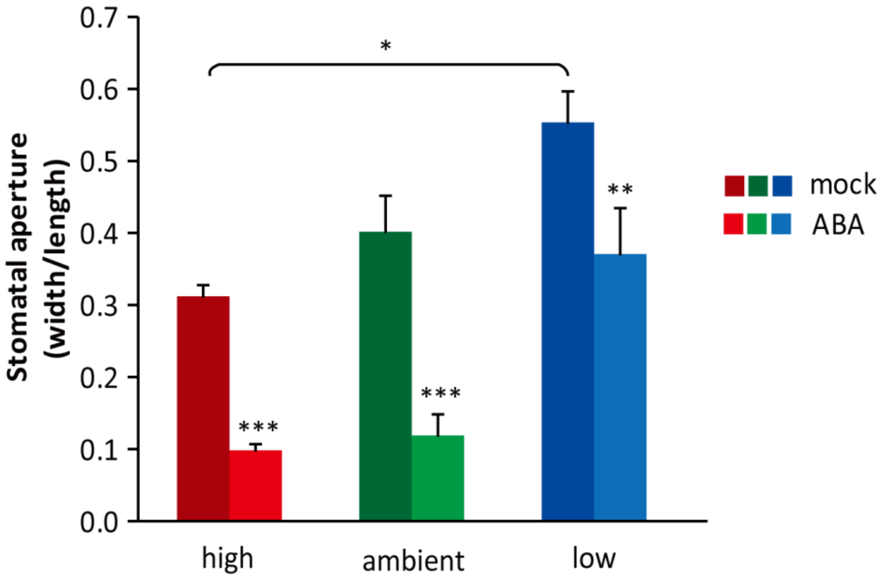

Fig. S4: The role of ABA signaling in Arabidopsis resistance to *Pst*. Growth of *Pst* in the ABA hypersensitive mutant *abi1-2*, wild-type Col-0 and the ABA deficient mutant *aba2-1* measured at 3 h, 2 days and 4 days after dip inoculation under the ambient CO_2 condition. Indicated are the averages of the log_{10}-transformed bacterial titer (±SD; per g of leaves) from eight biological replicates. Different letters indicate statistically significant differences between the genotypes at the specific time point (two-way ANOVA, Fisher's LSD test, $P<0.05$; ns, not significant). Indications above the brackets specify the interaction (Arabidopsis genotype × time) between the three Arabidopsis genotype (wild-type Col-0 and the mutants *abi1-2*, *aba2-1*) and time (3 hpi and 4 dpi) (***, $P<0.001$). The figure is representative of two independent experiments.

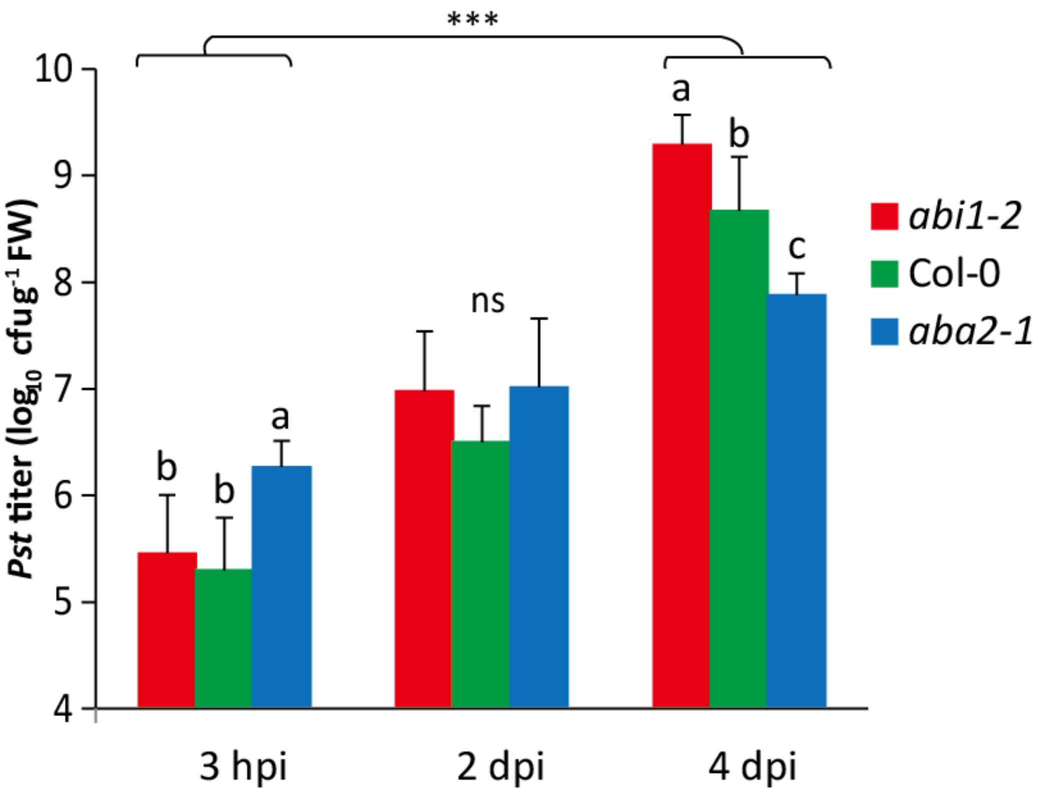

Supplement: Supplementary file 1 [file Image_1.pdf]
